# Special Issue “Mitochondria and Brain Disease”

**DOI:** 10.3390/biomedicines10081854

**Published:** 2022-08-01

**Authors:** Susana Cardoso

**Affiliations:** 1 Center for Neuroscience and Cell Biology (CNC), University of Coimbra, 3004-504 Coimbra, Portugal; susana.t.cardoso@gmail.com; 2 Institute for Interdisciplinary Research (IIIU), University of Coimbra, 3030-789 Coimbra, Portugal; 3 Center for Innovative Biomedicine and Biotechnology (CIBB), University of Coimbra, 3004-504 Coimbra, Portugal

We are pleased to present the first Special Issue (SI) of “Mitochondria and Brain Disease”. The scope of the present SI was to collect papers devoted to the multifaceted investigation of mitochondrial function and mitochondrial-directed interventions in the broad and heterogeneous field of brain diseases. Several renowned researchers contributed to this diverse collection, which includes five original research articles and eleven literature reviews. Topics addressed in this SI include the comprehensive discussion of mitochondrial function in early life stress (ELS)-affected brain [1], as well as in age-related neurodegenerative disorders such as Parkinson’s (PD) [2] and Alzheimer’s diseases (AD) [3]. In the same line, Leal and Martins [4] provide an updated review on the involvement of mitochondria–endoplasmic reticulum contact sites (MERCS) in neurodegenerative disorders, in particular AD. In turn, Onyango et al. [5] discuss the interaction between age-related mechanisms of disease (i.e., mitochondrial dysfunction, oxidative stress, defective autophagy, cellular senescence, etc.) and neuroinflammation in the pathogenesis of late-onset AD. A different perspective is presented by Bennett and Onyango [6] that discuss the involvement of mitochondria in age-related diseases from a thermodynamic point-of-view. In addition, a review manuscript by Brunetti et al. [7] discusses the role of defective mitochondrial proteostasis, namely due to the dysfunctional activity of pitrilysin metallopeptidase 1 (PITRM1), as a possible driving factor of several neurodegenerative conditions, particularly AD. Likely, another review manuscript by Lucini and Braun [8] discusses the role of mitochondria in TDP-43 proteinopathy and the involvement of TDP-43-mediated mitochondrial dysfunction in neurodegenerative diseases. In turn, the study of Kurokin et al. [9] shows that changes in lipid classes within mitochondria may correlate with the APP processing, i.e., whether it goes through the amyloidogenic or non- amyloidogenic pathway, and may compromise mitochondrial function. In the context of mental disorders, the review manuscript by Bressan and Kramer [10] presents a broad perspective on how mental disease relates to the different evolutionary strategies of men and women and to growth, metabolism, and mitochondria. In addition, Marques et al. [11] demonstrate that the loss of mitochondrial function is an early event implicated in bipolar disorder pathophysiology that might trigger neuronal changes and the modification of brain circuitry. The role of mitochondria in ischemic stroke and I/R injury is also discussed by Carinci et al. [12] who comprehensibly review the principal mitochondrial molecular mechanisms that function during the insults and present potential neuroprotective strategies targeting mitochondrial dysfunction and mitochondrial homeostasis. The importance of mitochondria in the cascade of events regulated by the IGF1/IGF1R signalling pathway is also described by Cardoso et al. [13]. In the same line, Lu et al. [14] demonstrate that mitochondrial function is improved in an animal model of epilepsy treated with medicinal plant-derived substances. Finally, this SI comprises two manuscripts that address the importance of mitochondria in oligodendrocyte function. One of those is an original study reporting that mitochondria is an important mediator in the loss of oligodendrocytes and myelin that characterizes Krabbe disease (KD) [15]. The other one discusses the role of mitochondria and endoplasmic reticulum in the malfunction of oligodendrocytes induced by harmful exogenous stimuli [16].

Given all these varied contributions, it is clear that mitochondria are powerful organelles that enable our existence, and the disruption of its function is theorized to have a causative role in several brain-related diseases. Importantly, these studies also highlight the therapeutic potential of rescuing mitochondrial integrity in such brain-related pathologies. There are still many important questions that remain unanswered, promising a great future for this field. 
